# Antibiotic Resistance to Critically Important Antimicrobials and Virulence Genes in *Enterococcus faecalis* Strains Isolated from Eurasian Griffon Vultures (*Gyps fulvus*) and Their Association with Mobile Genetic Elements

**DOI:** 10.3390/vetsci12111083

**Published:** 2025-11-14

**Authors:** Ana Isabel Vela, Carlos Serna, María Ugarte-Ruiz, Aránzazu Buendia, Almudena Casamayor, Johan Manuel Calderón Bernal, Lucas Domínguez, María Dolores Cid, José Francisco Fernández-Garayzábal

**Affiliations:** 1 Departamento de Sanidad Animal, Facultad de Veterinaria, Universidad Complutense, 28040 Madrid, Spain; 2 Centro de Vigilancia Sanitaria Veterinaria (VISAVET), Universidad Complutense, 28040 Madrid, Spain; 3 Facultad de Medicina Veterinaria y Zootecnia, Universidad Cooperativa de Colombia, Villavicencio 500001, Colombia

**Keywords:** vultures, microbiota, *Enterococcus faecalis*, antimicrobial resistance, virulence genes, mobile genetic elements, genotyping

## Abstract

The phenotypic resistance of 56 *Enterococcus faecalis* isolates from Eurasian griffon vultures was subjected to surveillance testing with the microdilution method using a standardized panel of antimicrobials. Isolates were also characterized by MLST. Additionally, the genome of 19 isolates with phenotypic resistance to linezolid, ciprofloxacin, chloramphenicol and/or high-level resistance to gentamicin were sequenced to determine their antimicrobial resistance (ARGs) and virulence-associated genes and to identify mobile genetic elements (MGEs). Most isolates (82.1%) exhibited non-wild-type phenotypes to six antimicrobial agents, and multidrug resistance (MDR) was detected in 34% of the isolates. Most MDR isolates (53%) belonged to ST16, ST116 and ST35. ARGs were localized on the chromosome as well as on various MGEs previously reported in human, food and livestock isolates, suggesting that vultures may acquire antibiotic-resistant bacteria (ARBs) and/or ARGs as a consequence of anthropogenic pollution. Overall, 22 virulence-associated genes encoding cell surface and secreted factors were identified, some of which were located on MGEs that also carried ARGs. The significant proportion of *E. faecalis* isolates recovered from vultures that exhibited MDR phenotypes and harbored MGEs carrying ARGs and virulence-associated genes is cause for concern, since vultures may act as spreaders of these genes to the environment, domestic animals and humans.

## Simple Summary

The purpose of this study was to investigate whether Eurasian griffon vultures could act as reservoirs of antimicrobial resistance genes (ARGs) and mobile genetic elements (MGEs) that determine the phenotypic resistance of *Enterococcus faecalis* isolates from these birds to various antimicrobials used in veterinary and human medicine. Additionally, the study aimed to characterize the ARGs, MGEs and virulence genes in the genome of a subset of isolates showing phenotypic resistance to the critically important antimicrobials linezolid, chloramphenicol, ciprofloxacin and gentamicin. Vultures were chosen because of their wide geographical distribution, their scavenging habits and their close proximity to humans. Most *E. faecalis* isolates (82.1%) exhibited resistance to six antimicrobials, indicating the widespread presence of resistant bacteria within this vulture population. Of particular concern was the detection of isolates resistant to linezolid, chloramphenicol, ciprofloxacin and gentamicin, considered clinically important in human medicine. Overall, a significant proportion of *E. faecalis* strains recovered from vultures were multidrug resistant (34%) harboring MGEs (plasmid replicons, transposons and composite transposons) that carried antimicrobial resistance and virulence-associated genes. These findings are cause for concern, since vultures may act as spreaders of these genes to the environment and even to other hosts.

## 1. Introduction

Antimicrobial resistance (AMR) is a complex problem that affects humans, animals and the environment. It is considered the greatest public and animal health threat of the 21st century [1] and requires a coordinated approach across different sectors to mitigate its impact [2]. Wildlife serves as a link between environmental, human and animal domains through direct and indirect contact, fecal contamination and shared environmental resources [3]. It is often referred to as a sentinel, reservoir and bridging host, facilitating the persistence of AMR in its habitats [4,5]. Although the role of wildlife has been highlighted with the application of the “One Health” concept to AMR, studies characterizing AMR in wild animals are not as exhaustive as those carried out with humans and livestock [4]. These studies show that antibiotic-resistant bacteria (ARBs) can be isolated from a multitude of wild animals across different geographical areas, including wild birds, mammals and reptiles [6,7,8,9]. In addition, because many wild animal species are highly mobile, they can play a crucial role in the transmission of ARBs over long distances [10]. Consequently, the role of wildlife in the dissemination of AMR might be underestimated [4,5], and further research in this area is needed to properly assess the public health implications of AMR in wild animals.

Previous studies have isolated various ARBs, such as *Escherichia coli*, *Salmonella* spp., *Campylobacter* spp. and *Enterococcus* spp., from vultures [11,12,13]. This species may serve as reservoirs of ARBs within their microbiomes with the potential to spread resistance genes over long distances and into new areas [6]. In particular, enterococci are considered indicator bacteria used to study the extent of AMR in populations owing to their ability to rapidly develop resistance mechanisms and further spread resistance and virulence genes [5,14]. Moreover, *Enterococcus faecalis* is one of the most common bacterial species in the cloaca and pharynx of Eurasian griffon vultures (*Gyps fulvus*) [15], the most widespread vulture across Europe, Asia and Africa (https://www.birdlife.org, accessed on 9 June 2025). Strains of *E. faecalis* isolated from vultures have been reported to carry clinically important resistance determinants and virulence traits [11,13].

One of the goals of the National Plan Against Antibiotic Resistance is to control the spread of resistant bacteria, especially those with transferable resistance mechanisms (https://www.resistenciaantibioticos.es/es, accessed on 20 September 2025). Horizontal gene transfer (HGT) involved in AMR can be mediated by different mobile genetic elements (MGEs) such as conjugative transposons (also called integrative and conjugative elements, ICE) or plasmids [16]. Both types of MGEs have been detected in enterococci isolated from different sources [17,18]. Nevertheless, information on the presence of MGEs among *E. faecalis* within the vulture microbiota is lacking. Thus, we hypothesize that MGEs will also gather in the guts and pharynges of Eurasian griffon vultures through the food chain. The purpose of this study was to investigate whether Eurasian griffon vultures could act as reservoirs of ARGS and MGEs. To this end, we determined the phenotypic resistance levels of cloacal and pharyngeal *E. faecalis* isolates from Eurasian griffon vultures to various antimicrobials used in veterinary and human medicine with a focus on characterizing ARGs and virulence genes in isolates exhibiting resistance to four critical antimicrobials: linezolid, chloramphenicol, ciprofloxacin and high-level resistance (HLR) to gentamicin.

## 2. Materials and Methods

### 2.1. Enterococcus Faecalis Isolates and Susceptibility Testing

The study focused on 56 *E. faecalis* isolates recovered from cloacal (*n* = 35) or pharyngeal (*n* = 21) samples of 45 Eurasian griffon (*Gyps fulvus*) vultures [15]. The *E. faecalis* isolates were subjected to surveillance testing with the microdilution method [19] using a standardized panel of antimicrobials (Sensititre EU Surveillance *Enterococcus* EUVENC Antimicrobial Susceptibility Testing Plates). In brief, inocula of the isolates were prepared in Muller–Hinton broth, adjusted to a 0.5 McFarland standard, and further diluted 1/220 in sterile distilled water. They were then deposited into each well of the microdilution plates, which were subsequently incubated at 37 °C for 24 h. *E. faecalis* ATCC 29212 and *Staphylococcus aureus* ATCC 29213 were used as control strains. The epidemiological cut-off (ECOFF) values (ECVs) used for the interpretation of the minimal inhibitory concentrations (MICs) of the isolates were in accordance with the guidelines of the European Committee for Antimicrobial Susceptibility Testing (https://www.eucast.org, accessed on 3 March 2025) and the European Decision 2020/1729 [20]. Based on the ECV, the isolates were classified into wild-type (WT, without phenotypically detectable resistance) and non-WT (with phenotypically detectable resistance) categories (Table 1). HLR to gentamicin was defined as strains exhibiting MICs > 500 μg/mL, in accordance with the recommendations of the Clinical and Laboratory Standards Institute [21]. Isolates with phenotypically detectable resistance to at least one antimicrobial in more than three different antimicrobial classes were classified as multidrug resistant (MDR). To assess the contribution of putative active efflux among the resistance mechanisms of gentamicin, erythromycin and ciprofloxacin in the 19 isolates, the MICs of these antimicrobials were determined in the presence or absence of the inhibitor reserpine (final concentration, 20 µg/mL; Sigma-Aldrich, St. Louis, MO, USA). The experiments were repeated three times. An efflux mechanism was inferred to be present when the antibiotic MICs in the presence of reserpine were at least four-fold lower than the corresponding MICs in the absence of this compound [22].

### 2.2. Multilocus Sequence Typing (MLST)

The 56 isolates were characterized using primers and conditions for PCR amplification of seven housekeeping gene fragments (*gdh*, *gyd*, *pst*S, *gki*, *aro*E, *xpt* and *yqi*L) included on the website of the *E. faecalis* MLST database (https://pubmlst.org/organisms/enterococcus-faecalis, accessed on 1 April 2025). The MLST alleles and resulting sequence types (STs) were assigned by submitting of the amplified sequences or respective allelic profiles to the *E. faecalis* MLST database. Genetic diversity (GD) was calculated as the ratio of the total number of STs to the total number of isolates [23]. The MLST profiles of *E. faecalis* available for download from the MLST website (https://pubmlst.org/organisms/enterococcus-faecalis) were used to generate a minimum-spanning tree with Phyloviz V2.0 and the goeBURST algorithm [24].

### 2.3. Whole-Genome Sequencing (WGS)

A total of 19 *E. faecalis* isolates were selected for WGS based on phenotypically detectable resistance to four antimicrobials (linezolid, ciprofloxacin, chloramphenicol and/or HLR to gentamicin) (Table S1). The genomic DNA of 13 isolates was sent to STAB-VIDA (Caparica, Portugal) for WGS. Genomic DNA of the isolates was extracted using the MagMax core kit (Applied Biosystems, Waltham, MA, USA) and the KingFisher Flex System automated extraction instrument (Thermo Fisher Scientific, Waltham, MA, USA) according to the manufacturer’s protocol. The concentration of genomic DNA was quantified and verified using the Qubit^®^ dsDNA BR Assay Kit (Thermo Fisher Scientific). The degree of genomic DNA degradation was evaluated through agarose gel electrophoresis. The sequencing libraries were prepared using the KAPA HyperPrep Library Preparation Kit (Roche, Basel, Switzerland) following the manufacturer’s recommended protocol and sequenced using the Illumina NovaSeq platform with the TrueSeq Library Prep Kit (Caprica; Portugal) (paired end 150 bp). Quality control of the raw data generated was performed using FastQC v0.11.9 (https://www.bioinformatics.babraham.ac.uk/projects/fastqc, accessed on 20 November 2024). Trimming and de novo assembly were carried out with CLC Genomics Workbench v12.0.3 (Qiagen, Venlo, Netherlands). All assemblies were performed with an automatic word size, a similarity fraction of 0.95, a length fraction of 0.95 and a minimum contig size of 500 bp. In addition, genomic DNA from six other isolates was sent to Secugen (Madrid, Spain) for WGS. Total DNA extraction and purification were performed with the MagAttract HMW DNA kit (Qiagen, Venlo, Netherlands). Subsequently, the quality and concentration of DNA were assessed using NanoDrop (Thermo Fisher Scientific, Waltham, MA, USA) and Qubit (Invitrogen; Thermo Fisher Scientific, Waltham, MA, USA) devices. Genomic libraries were prepared in accordance with the 1D native barcoding genomic DNA protocol using SKQ-LSK114 and SQK-NBD114.96 kits (Oxford Nanopore Technologies, Oxford, UK). Sequencing was performed using MinION equipment on a FLO-MIN114 vR10.4.1 flow cell at a speed of 400 bp/s (5 kHz). Long-read assemblies were carried out with Flye v2.9.1 [25] using default parameters. Assembly quality was assessed with QUAST v5.0.2 [26] and CheckM2 v1.1.0 [27]. Per-sample statistics are summarized in Table S2.

Raw Illumina and nanopore sequence data were deposited under project PRJNA1277192 (https://www.ebi.ac.uk/ena, accessed on 13 June 2025).

### 2.4. Bioinformatic Analysis of Whole-Genome Sequences

The assembled genomes were subjected to in silico screening for ARGs and virulence genes using the genomic tools ResFinder v4.0 and VirulenceFinder v2.0, respectively (https://www.genomicepidemiology.org, accessed on 15 Mach 2025), with the following cut-off values: a minimum of 90% coverage and 80% identity. ARGs were also screened against the Comprehensive Antibiotic Resistance Database (https://card.mcmaster.ca/analyze/rgi, accessed on 16 Mach 2025) using the default criteria (perfect and strict hits only) to supplement and confirm the ResFinder results. When both tools identified the same locus, duplicate entries were merged, and a single representative record was retained. LRE-Finder (https://www.genomicepidemiology.org; accessed on 16 Mach 2025) was used to detect 23S rRNA mutations and *optr*A, *cfr*, *cfr*(B), and *poxt*A genes encoding linezolid resistance in enterococci from whole-genome sequences [28]. All the outputs were integrated under the same non-redundant rule. Mobile Element Finder was used to identify the MGEs in the genomes (https://www.genomicepidemiology.org, accessed on 20 Mach 2025), while plasmid replicon genes, incompatibility groups and associated contigs were determined using PlasmidFinder 2.16 (https://www.genomicepidemiology.org, accessed on 20 March 2025) and MOBsuite v3.0.3 [29].

### 2.5. Phylogenetic Analysis

Mapping and whole-genome and core-genome alignment of the 19 *E. faecalis* genomes were performed using Snippy v4.6.0 software. A maximum likelihood tree based on the concatenated alignment of high-quality single-nucleotide polymorphisms (SNPs) was constructed with FastTree v2.1.10 [30] using the GTR + CAT models of nucleotide evolution, and the phylogenetic tree was visualized with iTOL v6.9.1.

### 2.6. Statistical Analysis

The association between AMR and MDR frequencies and the source (cloacal or pharyngeal samples) of the isolates was determined using Fisher’s exact test, with a significance threshold of *p* < 0.05. Bonferroni correction was applied for multiple comparisons. Statistical analyses were performed using Epi-InfoTM v7.2.5 software (Centers for Disease Control and Prevention; https://www.cdc.gov/epiinfo/esp/es_pc.html, accessed on 1 April 2025).

## 3. Results

### 3.1. Phenotypic Antimicrobial Resistance

The percentages of isolates showing non-WT phenotypes to the 12 antimicrobials tested are presented in Table 1. No significant differences were found between enterococci from cloacal and pharyngeal swabs for any of the 12 antimicrobials (*p >* 0.05). Most (82.1%) of the enterococcal isolates exhibited non-WT phenotypes to six antimicrobial agents (Table 2). The most prevalent non-WT phenotype was to tetracycline (82.1%), followed by erythromycin (58.9%). The non-WT rates for ciprofloxacin, gentamicin, chloramphenicol and linezolid ranged between 25.0% and 3.5%, whereas no non-WT phenotypes were found for the remaining six antibiotics (vancomycin, teicoplanin, daptomycin, quinupristin/dalfopristin, tigecycline and ampicillin). HLR to gentamicin was detected in 13 isolates.

The antimicrobial resistance profiles are presented in Table 2. MDR was detected in more than 30% of the isolates (*p >* 0.05; Table 2). MDR profiles were generally characterized by phenotypic resistance to gentamicin, tetracycline and erythromycin. The most common phenotypic resistance profile was erythromycin-tetracycline (ERY-TET, 25.0% of the isolates), followed by tetracycline (TET, 23.2% of the isolates).

### 3.2. Molecular Characterization of the Isolates

Overall, significant genetic heterogeneity (GD 0.55) was observed among the 56 *E. faecalis* isolates, which were classified into 31 STs after MLST analysis (Table 3). The most frequent genotypes were ST300, ST16 and ST648, represented by nine, five and four isolates, respectively. The other two STs included three isolates each (ST4 and ST116). Additionally, six STs were represented by two strains each (ST35, ST40, ST82, ST256, ST287 and ST706). The remaining STs were represented by single isolates.

The 19 MDR isolates also exhibited significant genetic diversity (GD 0.68) and were classified into 13 STs, all of them previously documented in the *E. faecalis* MLST database from different sources (Table 3). Three STs (ST16, ST116 and ST35) were detected in 52.6% of the MDR isolates (Table 3). Except for ST4, ST16 and ST82, the remaining 10 STs included only MDR isolates.

**Figure 1:**
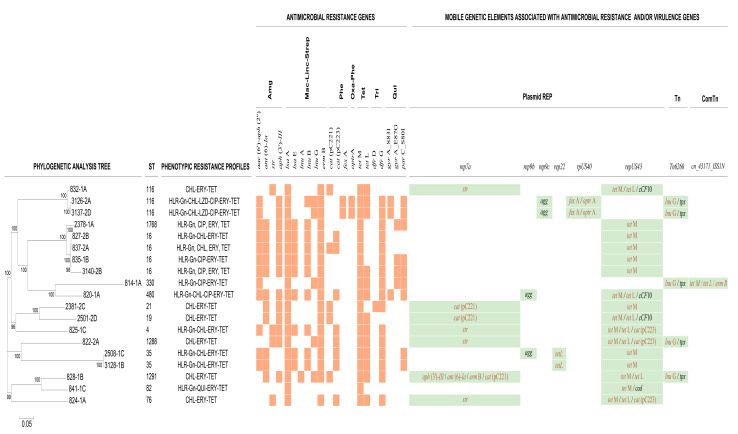
Phylogenetic tree generated from whole-genome SNP analysis of 19 non-wild-type vulture enterococci with resistance to ciprofloxacin, linezolid, chloramphenicol and/or high-level resistance to gentamicin. The figure shows the in silico identification of antimicrobial resistance genes and mobile genetic elements (MGEs). Except for isolates 822-2A, 824-1A, 825-1C, 828-1B and 835-1B, *repUS43* and *Tn6009* were found co-located with tetracycline resistance genes. Resistance genes associated with MGEs are indicated in orange, while virulence genes are shown in black. Abbreviations: Amg, Aminoglycoside; Mac-Linc-Strep, Macrolide-Lincosamide-Streptogramin; Phe, Phenicols; Oxa-Phe, Oxazolidinone-Phenicols; Tet, Tetracyclines; Tri; Trimethoprim; Qui, Quinolones; REP, replicon type; Tn, Transposon; ComTn, Composite Transposon.

Pairwise SNP differences among the 19 vulture *E. faecalis* with non-WT phenotype to ciprofloxacin, linezolid and chloramphenicol and/or with HLR to gentamicin are shown in Table S3. The minimum and maximum SNP differences were 0 and 17,168, respectively. The phylogenetic tree generated on the basis of whole-genome SNP (WG-SNP) revealed three small clades for the sequence types ST116 (3 isolates), ST16 (5 isolates) and ST35 (2 isolates), whereas the other STs were genetically diverse (Figure 1).

### 3.3. Antimicrobial Resistance Genes and Mobile Gene Elements (Plasmids, Transposons, and Integrative and Conjugative Elements)

Figure 1 shows the presence of ARGs detected in the genomes of the 19 isolates exhibiting a non-WT phenotype to linezolid, ciprofloxacin and chloramphenicol and/or with HLR to gentamicin. Twelve non-WT isolates to chloramphenicol carried *cat* genes encoding resistance to this antibiotic, *cat*(pC223) (58.3% isolates) and *cat*(pC221) (41.7%), whereas the two isolates showing phenotypic resistance to chloramphenicol and linezolid (3126-2A and 3137-2D) were found to contain genes encoding resistance to phenicols (*fex*A) and phenicols/oxazolidinones (*optr*A). The *aac*(6′)-*Ie-aph*(2″)-*Ia* gene conferring resistance to aminoglycosides was detected in 12 of 13 isolates with the HLR phenotype to gentamicin. Seven isolates with a non-WT phenotype to ciprofloxacin carried chromosomal point mutations in the quinolone resistance-determining regions (QRDRs) of the DNA gyrase subunit A (*gyr*A) and topoisomerase IV subunit A (*par*C) genes. Three amino acid mutations were detected in the *gyr*A gene, two at codon 83 (Ser to Tyr [*n* = 1] and Ser to Ile [*n* = 2]) and another at codon 87 (Glu to Gly [*n* = 4]), whereas a single amino acid mutation was found at *par*C codon 80 (Ser to Ile [*n* = 7]). This amino acid change at codon 80 in the *par*C gene was also detected in two WT isolates.

Genes conferring resistance to other antimicrobials included in the commercial panel used for phenotypic resistance screening were also identified. The *tet*M gene and *tet*M/*tet*L genes were found in 31.5% and 68.4% of the isolates with a non-WT phenotype to tetracycline, respectively (Figure 1). The *erm*B gene was identified in 94.7% of the isolates with the non-WT phenotype to erythromycin.

In addition, genes conferring resistance to other antimicrobials not included in the commercial panel used for phenotypic resistance screening were also identified (Table S1). Thus, the *ant*(6)-*Ia* and *str* genes, which confer resistance to streptomycin, were detected in 11 and four isolates, respectively, whereas the *aph*(3′)-*III* gene, which confers resistance to kanamycin, was detected in 13 isolates. The presence of the *dfr*G gene, which confers resistance to trimethoprim, was identified in the genomes of 16 isolates. The *lnu* (*lnu*A, *lnu*B and *lnu*G) genes and *lsa* (*lsa*A and *lsa*E) genes conferring resistance to lincomycin were also frequently detected.

The contribution of putative active efflux was demonstrated by an antibiotic/reserpine (A/R) MIC test in only one isolate. This isolate showed a non-WT phenotype to ciprofloxacin and erythromycin and HLR to gentamicin, yet no resistance genes or chromosomal point mutations in the *gyr*A and *parC* genes were detected in its genome. The isolate had lower MICs for these antibiotics after exposure to reserpine compared to MICs in the absence of this compound (MICs of 16 µg/mL vs. 1024 µg/mL, 0.25 µg/mL vs. 16 µg/mL and <1 µg/mL vs. >128 µg/mL for gentamicin, ciprofloxacin and erythromycin, respectively). The *efr*A and *erf*B genes, which encode efflux pumps for different antimicrobials, were detected in the genome of this isolate.

Four plasmid replicon types harboring ARGs were detected in the 19 isolates (Figure 1, Table S1). The most prevalent was *repUS43*, which was detected in 16 isolates (89.5%). This replicon was found to be chromosomally integrated and co-located on the same contig with *tet*M (10 genomes) or *tet*M/*tem*L (three genomes; 820-1A, 828-1B and 832-1A) genes in 13 *E. faecalis* isolates exhibiting a non-WT phenotype to tetracycline. In addition, *cat* genes, together with *tet*M/*tem*L, were bound to *repUS43* in three genomes (822-2A, 824-1A and 825-1C). The *tet*L gene was also associated with the *rep22*-type plasmid, whereas the *str, aph*(3′)-*III*, *ant*(6)-*Ia*, *erm*B and *cat* genes were associated with *rep7a* (Table S1, Figure 1). The *repUS40* replicon was associated with the *optr*A and *fex*A genes in both isolates exhibiting non-WT phenotypes to linezolid and chloramphenicol.

In addition, other MGEs, such as transposons (Tns) and composite transposons (ComTns), were also associated with ARGs. *Tn*6009 was detected in 57.9% of the isolates. This MGE was associated with *repUS43* and tetracycline resistance genes. The *lnu*G gene was found to be embedded in *Tn6260* in five genomes, whereas ComTn, *cn_43171_ISS1N*, was linked to tetracycline (*tet*M, *tet*L) and macrolide (*erm*B) resistance genes in one genome.

### 3.4. Virulence Factors

A total of 22 virulence factors were found among the 19 genomes (Table 4). All isolates harbored genes encoding sex pheromone-associated proteins (*cad*, *cCF*10, *cam*E, *cOB*1), protection against oxidative stress (*tp*x), cell wall adhesion (*efa*A*fs*), biofilm-associated pili (*ebp*A, *ebp*B, *ebp*C) and the cell wall anchor surface protein sortase A (*srt*A). Genes associated with the cytolysin toxin (*cyl*A, *cyl*B, *cyl*L and *cyl*M) were identified in seven isolates (12.5%). Other virulence genes identified in most of the 19 isolates were *elr*A (Rgg-like regulator gene associated with macrophage persistence; 94.7% of the isolates), *agg* (aggregation substance; 57.9% of the isolates), *frs*B (quorum-sensing regulator; 63.2% of the isolates), *gel*E (gelatinase toxin; 63.2% of the isolates), *ace* (collagen adhesion precursor; 78.9% of the isolates), *hyl*A and *hyl*B (hyaluronidase genes; 73.7% and 47.4% of the isolates, respectively). The *espfs* gene (enterococcal surface protein) was identified in 5.3% of the isolates.

Some virulence genes were associated with MGEs (Figure 1). Thus, the *cCF10* and *cad* genes were bound to *repUS43* in three and one isolates, respectively. In addition, the *agg* gene was bound to replicons *rep9b* and *rep9c*, in two isolates each, and the *tpx* gene was bound to transposon *Tn6260*, in five isolates.

## 4. Discussion

The impact of wild animals as reservoirs of AMR and ARGs, which can later be disseminated among different hosts, and into the environment [3], has received increasing attention from the “One Health” perspective in recent years. Thus, the present study investigated the prevalence of AMR in a collection of 56 *E. faecalis* isolates from the pharynx and cloaca of Eurasian griffon vultures. These birds were chosen because of their wide geographical distribution and their scavenging habits, and because they live in close proximity to humans. In addition, enterococci are widely considered key microbiota indicators for tracing the spread and evolution of MDR bacteria in the environment and in wildlife [5]; furthermore, *E. faecalis* is a common inhabitant of the cloacal and pharyngeal microbiota of Eurasian griffon vultures [15].

The high percentage of *E. faecalis* isolates (82.1%) exhibiting non-WT phenotypes to six antimicrobials, indicates the widespread presence of AMR in isolates of *E. faecalis*, which is consistent with the high rates of AMR and MDR in enterococci detected in various wild bird species, including vultures [11,31,32]. The highest resistance rates were detected for erythromycin and tetracycline (Table 1), and one third of the isolates were phenotypically MDR (Table 2). Accordingly, the most common AMR profile observed was resistance to erythromycin and tetracycline (Table 2), which is in line with previous studies on *E. faecalis* collected from different animal and human sources [9,33]. Linezolid, chloramphenicol, ciprofloxacin and HLR to gentamicin are considered clinically important antimicrobials in human medicine [34]. Thus, of special concern is that over half (63%; 12 out of 19 isolates) of the MRD isolates exhibited a non-WT phenotype to at least two of these antimicrobials (Table 2). *E. faecalis* strains with a resistant phenotype to any of these four antimicrobial agents have been reported in various wild bird species, including vultures [7,13,31].

The 31 STs identified after MLST analysis indicate high genetic heterogeneity (GD 0.55) among the 56 *E. faecalis* isolates. This genetic diversity was similar between non-MRD and MRD *E. faecalis* isolates (GD 0.56 and GD 0.68, respectively), although MRD and non-MRD isolates differed in the STs identified in each group. Most STs identified in MRD isolates (10 out of 13) were detected exclusively in these isolates. Similarly, 19 of the 21 STs identified in the non-MRS isolates were detected in only these isolates (Table 3). Moreover, ST16 and ST116 accounted for almost 40% of the MRD isolates, whereas only one non-MRD isolate belonged to either of these two genotypes (Table 3). These results suggest a different genetic background for the *E. faecalis* populations of MRD and non-MRD isolates. Additionally, 17 of the 19 sequenced *E. faecalis* isolates exhibited SNP differences greater than 100, which exceeds the proposed cut-off value for considering isolates as clonal [35]. Thus, the genetic diversity observed by MLST suggests that the high rates of AMR detected in these *E. faecalis* isolates are more likely a consequence of exposure to multiple strains rather than the clonal spread of resistant isolates.

MLST enables the comparison of molecular typing data of a particular pathogen against those in a large central database, providing an overview of the distribution of its genetic lineages. Based on the information available on the *E. faecalis* MLST database website (https://pubmlst.org/organisms/enterococcus-faecalis, accessed on 1 April 2025), all STs identified in vultures in this study have been previously detected in animals, humans, foods and the environment, indicating the recirculation of these genotypes across different hosts and environments (Table 3). Human activities and food-animal production can contribute through various pathways to the development and spread of ARBs or ARGs. The feeding habits of Eurasian griffon vultures can also contribute to the acquisition of ARBs. These birds feed almost exclusively on carrion, primarily mammals from farming animals that have died from disease or accidents and are discarded at predictable sites that represent an abundant food source [36]. Although in the present study we have not investigated antimicrobial resistance in *E. faecalis* strains isolated from livestock animals or the environment near vulture feeding points, it is plausible to speculate that the concentration of vultures at these feeding sites may have facilitated their exposure to active antimicrobial residues that are present in the carcasses of medicated livestock [37]. Thus, the direct feeding of livestock carcasses treated with erythromycin and tetracycline, both widely used in veterinary medicine in Spain (https://www.aemps.gob.es/, accessed on 15 June 2025) could explain the high rates of *E. faecalis* isolates with the non-WT phenotype to these antimicrobials (Table 2). Also, vultures living in contact with anthropogenically impacted areas could be colonized by ARB strains, likely selected by antimicrobial agents used in humans [38]. This could explain the detection of *E. faecalis* isolates with a non-WT phenotype to antimicrobials such as linezolid, which is authorized only for human use [34]. Moreover, once ARBs are introduced into wildlife, they can persist for extended periods even in the complete absence of selection pressure from antimicrobial agents [39]. This persistence could explain the relatively high rates of *E. faecalis* isolates with a non-WT phenotype to chloramphenicol (Table 1), despite its use being banned in humans, pets and non-food-producing animals in the EU since the mid-1990s (https://www.aemps.gob.es/, accessed on 15 June 2025, [40]).

A subset of 19 selected isolates exhibiting non-WT phenotypes to the clinically important antimicrobials linezolid, chloramphenicol and ciprofloxacin and with HLR to gentamicin were sequenced. An in-depth analysis of their genetic resistance mechanisms was performed to better understand their AMR mechanisms. Several ARGs that have been detected in a wide variety of bacteria [40,41], such as the *cat*(pC221) and *cat*(pC223) genes encoding resistance to chloramphenicol and the *aac*(6′)-*Ie-aph*(2″)-*Ia* gene conferring HLR to gentamicin were identified in the genomes of the *E. faecalis* isolates (Table S1). Also, isolates with a non-WT phenotype to chloramphenicol and linezolid carried the *optr*A and *fex*A genes (Table S1), which are the main mechanisms of resistance of *Enterococcus* to oxazolidinone and phenicol antimicrobials [42]. ARGs to other antimicrobials included in the commercial panel that exhibited non-WT phenotypes were also identified. Thus, the non-WT phenotype to erythromycin and tetracycline was associated with the detection of the *emr*B gene (94.7% isolates) and the *tet*M (31.5%) or *tet*M/*tet*L (68.4%) genes, which were the most prevalent resistance genes among erythromycin- and tetracycline-resistant enterococci [7,31]. Seven of the eight isolates with a non-WT phenotype to ciprofloxacin and MICs of ≥16 µg/mL exhibited amino acid changes in the GyrA (S83I or S83Y, and E87G) and ParC (S80I) proteins, which have been previously reported in ciprofloxacin-resistant *E. faecalis* isolates [43,44].

We also identified other genes associated with resistance to antimicrobials not included in the commercial panel, such as the *aph*(3′)-*III* gene, which confers resistance to kanamycin and amikacin, and the *ant*(6)-*Ia* and *str* genes, which are associated with resistance to streptomycin [45,46]. However, phenotypic resistance to these antimicrobials was not tested in this study. The *efr*A, *erf*B and *lsa* genes, encoding efflux pumps for different antimicrobials in *E. faecalis* isolates [47], were detected in the genome of one isolate (841-1C) with a non-WT phenotype to ciprofloxacin and erythromycin and with HLR to gentamicin but without resistance genes or chromosomal point mutations in the *gyr*A and *par*C genes. The decrease in the MIC values for these antimicrobials by six- to seven-fold in the presence of reserpine versus in the absence of this compound suggests that antibiotic efflux pumps are involved in resistance to these antimicrobials in this isolate [48]. Although the efflux pump system has not been involved in erythromycin resistance in enterococci, it has been reported in other Gram-positive bacteria, such as staphylococci [49].

In addition to the different ARGs described above, MGEs, including plasmid replicons and transposons, which play a key role in the acquisition and dissemination of ARGs via HGT [17,18], were identified among the *E. faecalis* vulture isolates (Table S1, Figure 1), suggesting the potential of these isolates to acquire and transfer AMR. Remarkably, resistance genes to chloramphenicol, linezolid, tetracycline, erythromycin, streptomycin and kanamycin more frequently co-existed in different replicons and transposons (Figure 1; Table S1). We also found different MGEs carrying the same ARG as well as different ARGs carried by the same MGE, which could increase their dissemination potential. Thus, genes conferring resistance to different classes of antimicrobial agents were found in six isolates co-located with a plasmid replicon on the same contig (31.6%; Table S1): three isolates carried the *tet*M, *tet*L and *cat*(pC223) genes located in tandem close to the plasmid replicon *repUS43;* two isolates carried the *fex*A and *optr*A genes on the *repUS40* replicon; and one isolate carried the *ant*(6)-*Ia*, *aph*(3′)-*III*, *erm*B and *cat*(pC221) genes on the same contig as the *rep7a* replicon. All replicons and transposons identified in the *E. faecalis* isolates and described above have been previously reported in this and other enterococcal species isolated from humans, foods and livestock [18,44,50,51,52]. The acquisition of ARGs carried by MGEs can lead to the establishment of multidrug resistance [53]. In this context, the detection of different MGEs in the 19 sequenced isolates could explain why one-third of the 56 isolates were MDR (Table S1; Figure 1).

Most enterococci are not virulent and are considered relatively harmless, with limited potential to cause human infection. However, they have also been identified as nosocomial opportunistic pathogens with increased resistance to antimicrobial agents [54]. The 19 isolates investigated in this study harbored different virulence-related genes, such as those related to sex pheromone-associated proteins, protection against oxidative stress, cell wall adhesion, biofilm formation and macrophage persistence (Table 4), all of which are involved in enterococcal pathogenicity [55]. Of special concern is the detection of genes associated with cytolysin production (*cyl*A, *cyl*B, *cyl*L and *cyl*M) in seven of the 19 sequenced isolates, which is related to increased severity of infection in humans [55]. Thus, the presence of various virulence-associated genes in all *E. faecalis* vulture isolates suggests their possible pathogenic potential. Moreover, some MEGs, such as the replicon *repUS43* and the transposon *Tn6260*, carried both virulence genes and ARGs simultaneously. The co-presence of virulence genes and ARGs in the same MGEs could facilitate their spread to other bacteria within the pharyngeal and intestinal microbiota of vultures, which could represent an additional risk of pathogen transmission to exposed domestic animals and humans. In this sense, further investigations are needed to determine the impact of these commensal enterococci on human and animal health, particularly regarding the spread of antibiotic resistance and virulence.

## 5. Conclusions

Overall, the results of this study show that a significant proportion of *E. faecalis* strains recovered from cloacal or pharyngeal samples of vultures were phenotypically MDR and harbored MGEs (plasmid replicons, transposons and composite transposons) that carried AMR and/or virulence-associated genes. These findings are cause for concern, since vultures may act as spreaders of virulence and antimicrobial resistance genes to the environment and even to other hosts.

## Figures and Tables

**Table 1:** Number of *E. faecalis* with non-wild-type phenotypes to each of the 12 antimicrobial agents tested.

Class	Antimicrobial	Nº of Non-WT Isolates (%)
β-Lactam	AMP	0
Quinolone	CIP	8 (14.3)
Macrolide	ERY	33 (58.9)
Aminoglycoside	GEN	14 * (25.0)
Liopeptide	DAP	0
Tetracycline	TET	46 (82.1)
	TGC	0
Phenicol	CHL	14 (25.0)
Oxazolidinone	LZD	2 (3.6)
Streptogramin	SYN	0
Glycopeptide	TEI	0
	VAN	0

* HLR-Gn was detected in 13 of the 14 isolates. Abbreviations: non-WT, non-wild type; AMP, ampicillin; CIP, ciprofloxacin; ERY, erythromycin; GEN, gentamicin; DAP, daptomycin; TET, tetracycline; TGC, tigecycline; CHL, chloramphenicol; LZD, linezolid; SYN, quinupristin/dalfopristin; TEI, teicoplanin; VAN, vancomycin.

**Table 2:** Antimicrobial resistance profiles of *E. faecalis* isolates from Eurasian griffon vultures.

Antimicrobial Resistance Profile	Nº Isolates Showing the Antimicrobial Resistance Profile (%)
WT isolates	10 (17.9)
TET	**13 (23.2) ^1^**
ERY-TET	**14 (25.0) ^1^**
GEN-ERY-TET ^2^	2 (3.6)
CHL- ERY -TE T ^2^	5 (8.9)
GEN-CIP-TET ^2^	1 (1.8)
GEN-CIP-ERY-TET ^2^	4 (7.1)
GEN -CHL- ERY- TE T ^2^	4 (7.1)
GEN-CIP-CHL-ERY-TET ^2^	1 (1.8)
GEN-CIP-CHL-LZD-ERY-TET ^2^	2 (3.6)
Total non-WT isolates	46 (82.1)
Total MDR isolates	19 (33.9)

^1^ Most frequent profiles are indicated in bold. ^2^ Multidrug resistance phenotypes are underlined. Abbreviations: WT, wild type, non-WT, non-wild type; MDR, multidrug resistant; CIP, ciprofloxacin; ERY, erythromycin; GEN, gentamicin; TET, tetracycline; CHL, chloramphenicol; LZD, linezolid.

**Table 3:** Multilocus sequence types of the 56 *E. faecalis* isolates from Eurasian griffon vultures and their relationship to source recorded in the MLST *E. faecalis* database (https://pubmlst.org/organisms/enterococcus-faecalis, accessed on 1 April 2025).

ST	Nº Vulture Isolates			Nº Isolates *of E. faecalis* in the MLST Database					
	MDR	Non-MDR	Total	Animals *	Human	Foods	Environment	Unknown	Total
4	1	2	3	12	1			1	14
7		1	1	1					1
9		1	1	22		2			24
16	4	1	5	68	1			11	80
19	1		1	7	4	7		3	21
21	1		1	41	23	22		4	90
35	2		2		2				2
40		2	2	15	42	8	3	6	74
59		1	1	6	4			1	11
76	1		1	1	1			3	5
82	1	1	2	13	1	25		2	41
116	3		3	9	8	1	1	1	20
200		1	1	1					1
256		2	2	6	1		3	3	13
268		1	1		1				1
287		2	2		4		1		5
300		9	9	4	1				5
330	1		1	3	3			3	9
441		1	1		1				1
480	1		1	11	4			1	16
631		1	1	10	1				11
648		4	4	4	1				5
699		1	1	1					1
706		2	2	1					1
860		1	1			1			1
1287		1	1	2			1		3
1288	1		1	5					5
1291	1		1			1			1
1600		1	1			1			1
1768	1		1			1			1
1875		1	1	1					1

* Chicken, pets, livestock and wildlife animals. Abbreviations: MDR; multidrug resistant.

**Table 4:** Virulence-associated genes identified in the genome of 19 *E. faecalis* isolated from Eurasian griffon vultures.

Isolate	Virulence-Associated Genes											
	Cell Surface Virulence Factors				Secreted Virulence Factors							
	*elr*A	*ace*	*agg*	*espfs*	*cyl*A	*cyl*B	*cyl*L	*cyl*M	*fsr*B	*gel*E	*hyl*A	*hyl*B
832-1A												
3126-2A												
3137-2D												
2378-1A												
827-2B												
837-2A												
835-1B												
3140-2B												
814-1A												
820-1A												
2381-2C												
2501-2D												
825-1C												
822-2A												
2508-1C												
3128-1B												
828-1B												
841-1C												
824-1A												

*stra*A, *ebp*A, *ebp*B, *ebp*C, *efa*A*fs*, *cCF*10, *cOB*1, *cad*, *cam*E and *tpx* were detected in the genomes of the 19 isolates.

## Data Availability

The genomic sequencing data generated throughout this research have been deposited under project accession number PRJNA1277192, publicly available in the National Center for Biotechnology Information (NCBI) Database.

## Supplementary Materials

The following supporting information can be downloaded at: https://www.mdpi.com/article/10.3390/vetsci12111083/s1, Table S1: Phenotypic, genomic antimicrobial resistance and mobile genetic elements associated with antimicrobial resistance genes in 19 *E. faecalis* isolates investigated in this study; Table S2: Assembly statistics for *E. faecalis* genomes sequenced in this study; Table S3: Pairwise SNP differences between *E. faecalis* isolates with a non-wild-type phenotype to ciprofloxacin, linezolid, chloramphenicol and/or high-level resistance to gentamycin.

## Abbreviations

The following abbreviations are used in this manuscript:

AMR	Antimicrobial resistance
ARBs	Antibiotic-resistant bacteria
ARGs	AMR genes
HGT	Horizontal gene transfer
MGEs	Mobile genetic elements
HLR	High-level resistance
ECOFF	Epidemiological cut-off
ECVs	Epidemiological cut-off F values
MICs	Minimal inhibitory concentrations
WT	Wild type
MDR	Multidrug resistant
MLST	Multilocus sequence typing
STs	Sequence types
GD	Genetic diversity
CDC	Center for Disease Control and Prevention
WGS	Whole-genome sequencing
SNPs	Single-nucleotide polymorphisms
TET	Tetracycline
ERY-TET	Erythromycin-tetracycline
A/R	Antibiotic/reserpine
Tns	Transposons
ComTns	Composite transposons
