# Supplementary material for: Antibiotic Resistance to Critically Important Antimicrobials and Virulence Genes in Enterococcus faecalis Strains Isolated from Eurasian Griffon Vultures (Gyps fulvus) and Their Association with Mobile Genetic Elements
Source: Vet Sci. 2025 Nov 14;12(11):1083. doi: 10.3390/vetsci12111083 (PMC12656748; doi:10.3390/vetsci12111083)
Supplement: Supplementary file 1 [file vetsci-12-01083-s001.zip › Table S1.pdf]

Table S1: Phenotypic, genomic antimicrobial resistance and mobile genetic elements associated with antimicrobial resistance genes in 19 *E.* faecalis isolates investigated in this study.

Isolate	ST	Phenotypic AMR patter	AMR genes[a]	QRDRs	Plasmid REP	Tn	ComTn	MGEs associated with ARGs
814-1A	330	HLR-Gn, CIP, ERY, TET	*aac*(6')-*aph*(2''), *lnu*G, *erm*B, *tet*M, *tet*L, *dfr*G	*gyr*A_E87G and *par*C_S80I	*repUS43*	*Tn6260*	*cn_43171_ISSN*	*tet*M/*tet*L/*ermB* on *cn_43171_ISSN*, *lnu*G on *Tn6260*
820-1A	480	HLR-Gn, CHL, CIP, ERY, TET	*aac*(6')-*aph*(2''), *ant*(6)-*Ia*, *aph*(3')-*III*, *lsa*E, *lnu*B, *erm*B, *cat*(pC223), *tet*M, *tet*L, *dfr*G	*gyr*A_S83Y and *par*C_S80I	*repUS43*	*Tn6009*		*tet*M/*tet*L on *repUS43*/*Tn6009*
822-2A	1288	CHL, ERY, TET	*str*, *aph*(3')-*III*, *cat*(pC223), *lnu*G, *erm*B, *tet*M, *tet*L, *dfr*G		*rep7a*, *repUS43*	*Tn6260*		*str* on *rep7a*, *tet*M/*tet*L/*cat*(pC223) on *repUS43*, *lnu*G on *Tn6260*
824-1A	76	CHL, ERY, TET	*str*, *cat*(pC223), *erm*B, *tet*M, *tet*L, *dfr*G		*rep7a*, *repUS43*			*str* on *rep7a*, *tet*M/*tet*L/*cat*(pC223) on *repUS43*
825-1C	4	HLR-Gn, CHL, ERY, TET	*aac*(6')-*aph*(2''), *str*, *aph*(3')-*III*, *lsa*E, *lnu*B, *erm*B, *cat*(pC223), *tet*M, *tet*L, *dfr*G		*rep7a*, *repUS43*			*str* on *rep7a*, *tet*M/*tet*L/*cat*(pC223) on *repUS43*
827-2B	16	HLR-Gn, CHL, ERY, TET	*aac*(6')-*aph*(2''), *ant*(6)-*Ia*, *aph*(3')-*III*, *lnu*B, *lsa*E, *erm*B, *cat*(pC223), *tet*M, *dfr*G		*repUS43*	*Tn6009*		*tet*M on *repUS43*/*Tn6009*
828-1B	1291	CHL, ERY, TET	*ant*(6)-*Ia*, *aph*(3')-*III*, *cat*(pC221), *lnu*A. *lnu*G, *erm*B, *tet*M, *tet*L, *dfr*G		*rep7a*, *repUS43*	*Tn6260*		*aph*(3')-*III*/*ant*(6)-*Ia*/*cat*(pC221)/*erm*B on *rep7a*, *tet*M/*tet*L on *repUS43*, *lnu*G on *Tn6260*
832-1A	116	CHL, ERY, TET	*str*, *cat*(pC221), *erm*B, *tet*M, *tet*L		*rep7a*, *repUS43*	*Tn6009*		*str* on *rep7a*, *tet*M/*tet*L on *repUS43*/*Tn6009*
835-1B	16	HLR-Gn, CIP, ERY, TET	*aac*(6')-*aph*(2''), *ant*(6)-*Ia*, *aph*(3')-*III*, *lsa*E, *lnu*B, *erm*B, *tet*M, *dfr*G	*gyrA*_E87G and *par*C_S80I	*repUS43*	*Tn6009*		*tet*M on *repUS43*/*Tn6009*
837-2A	16	HLR-Gn, CHL, ERY, TET	*aac*(6')-*aph*(2''), *ant*(6)-*Ia*, *aph*(3')-*III*, *cat*(pC223), *lsa*E, *lnu*B, *erm*B, *tet*M, *dfr*G		*repUS43*	*Tn6009*		*tet*M on *repUS43*/*Tn6009*

841-1C	82	HLR-Gn, QUI, ERY, TET	*tet*M		*repUS43*	*Tn6009*	*tet*M on *repUS43*/*Tn6009*
2378-1A	1768	HLR-Gn, CIP, ERY, TET	*aac*(6')-*aph*(2''), *ant*(6)-*Ia*, *aph*(3')-*III*, *lsa*E, *lnu*B, *erm*B, *tet*M, *dfr*G	*gyr*A_E87G and *par*C_S80I	*repUS43*	*Tn6009*	*tet*M on *repUS43*/*Tn6009*
2381-2C	21	CHL, ERY, TET	*ant*(6)-*Ia*, *aph*(3')-*III*, *cat*(pC221), *erm*B, *tetM*, *dfrD*, *dfrG*		*rep7a*, *repUS43*	*Tn6009*	*cat*(pC221) on *rep7a*, *tet**M*** *repUS43*/*Tn6009*
2501-2D	19	CHL, ERY, TET	*ant*(6)-*Ia*, *aph(3'*)-*III*, *cat*(pC221), *ermB*, *tet*M, *tet*L		*rep7a*, *repUS43*	*Tn6009*	*cat*(pC221) on *rep7a*, *tet*M on *repUS43*/*Tn6009*
2508-1C	35	HLR-Gn, CHL, ERY, TET	*aac*(6')-*aph*(2''), *ant*(6)-*Ia*, *cat*(pC223), *lsa*E, *lnu*B, *erm*B, *tet*M, *tet*L, *dfr*G	*par*C_S80I	*rep22*, *repUS43*	*Tn6009*	*tet*L on *rep22*, *tet*M on *repUS43*/*Tn6009*
3126-2A	116	HLR-Gn, CHL, LZD, CIP, ERY, TET	*aac*(6')-*aph*(2''), *aph*(3')-*III*, *fex*A, *optr*A, *lnu*B, *lnu*G, *erm*B, *tet*M, *tet*L, *dfr*G	*gyr*A_S83I and *par*C_S80I	*rpUS40*	*Tn6260*	*fex*A/*optr*A on *repUS40*, *lnu*G on *Tn6260*
3128-1B	35	HLR-Gn, CHL, ERY, TET	*aac*(6')-*aph*(2''), *ant*(6)-*Ia*, *cat*(pC223), *lsa*E, *lnu*B, *erm*B, *tet*M, *tet*L, *dfr*G	*par*C_S80I	*rep22*, *repUS43*	*Tn6009*	*tet*L on *rep22*, *tetM* on *repUS43*/*Tn6009*
3137-2D	116	HLR-Gn, CHL, LZD, CIP, ERY, TET	*aac*(6')-*aph*(2''), *aph*(3')-*III*, *fex*A, *optr*A, *lnuG*, *ermB*, *tetM*, *tetL*, *dfrG*	*gyr*A_S83I and *par*C_S80I	*rpUS40*	*Tn6260*	*fex*A/*optr*A on *repUS40*, *lnu*G on *Tn6260*
3140-2B	16	HLR-Gn, CIP, ERY, TET	*aac*(6')-*aph*(2''), *ant*(6)-*Ia*, *aph*(3')-*III*, *lsa*E, *lnu*B, *erm*B, *tet*M, *tet*L, *dfr*G	*gyr*A_E87G and *par*C_S80I	*rep22*, *repUS43*	*Tn6009*	*tet*M on *repUS43*/*Tn6009*

[a] *lsaA* gene was present in all isolates. Abbreviations: AMR, Antimicrobial resistance; REP, replicon type; Tn, Transposon; ComTn, Composite Transposon; CIP, ciprofloxacin; ERY, erythromycin; HLR-Gn, high level resistance to gentamicin; TET, tetracycline; CHL, chloramphenicol; LZD, linezolid; QRDRs, quinolone resistance determinant regions; MEGs, mobile genetic elements; ARGs, antimicrobial resistance genes.
