# Supplementary material for: Antibiotic Resistance to Critically Important Antimicrobials and Virulence Genes in Enterococcus faecalis Strains Isolated from Eurasian Griffon Vultures (Gyps fulvus) and Their Association with Mobile Genetic Elements
Source: Vet Sci. 2025 Nov 14;12(11):1083. doi: 10.3390/vetsci12111083 (PMC12656748; doi:10.3390/vetsci12111083)
Supplement: Supplementary file 1 [file vetsci-12-01083-s001.zip › Table S2.pdf]

Table S2: Assembly statistics for *E. faecalis* genomes sequenced in this study.

Isolate	Contigs	Total length	GC (%)	N50	Completeness	Contamination
814_1A	7	3094665	37.37	2098973	100	0.3
820_1A	47	2859131	37.47	164183	99.99	0.23
822_2A	27	2859557	37.5	257043	100	0.1
824_1A	20	2761309	37.59	225419	100	0.05
825_1C	28	3023470	37.13	281218	100	0.16
827_2B	50	2873750	37.33	180624	100	0.1
828_1B	38	2882117	37.46	191169	100	0.32
832_1A	56	2815613	37.58	175946	100	0.05
835_1B	1	2910564	37.41	2910564	100	0.12
837_2A	37	2874162	37.33	391478	100	0.1
841_1C	5	3222021	37.06	3169282	100	0.38
2378_1A	1	2998808	37.31	2998808	100	0.16
2381_2C	24	2513553	37.59	259100	87.42	0.12
2501_2D	55	2969246	37.35	160930	100	0.3
2508_1C	30	3025010	37.26	274891	100	0.5
3126_2A	49	2911294	37.45	257413	100	0.12
3128_1B	39	2956601	37.35	169582	100	0.14
3137_1D	5	2951173	37.5	2722587	100	0.12
3140_2B	4	2863766	37.46	1810654	100	0.11
