# Supplementary material for: Antibiotic Resistance to Critically Important Antimicrobials and Virulence Genes in Enterococcus faecalis Strains Isolated from Eurasian Griffon Vultures (Gyps fulvus) and Their Association with Mobile Genetic Elements
Source: Vet Sci. 2025 Nov 14;12(11):1083. doi: 10.3390/vetsci12111083 (PMC12656748; doi:10.3390/vetsci12111083)
Supplement: Supplementary file 1 [file vetsci-12-01083-s001.zip › Table S3.pdf]

Table S3: Pairwise SNP differences between *E. faecalis* isolates with a non-wide type to ciprofloxacin, linezolid, chloramphenicol and/or high-level resistance to gentamycin.

**Reference**	**2378-1A**	**2381-2C**	**2501-2D**	**2508-1C**	**3126-2A**	**3128-1B**	**3137-2D**	**3140-2B**	**814-1A**	**820-1A**	**822-2A**	**824-1A**	**825-1C**	**827-2B**	**828-1B**	**832-1A**	**835-1B**	**837-2A**	**841-1C**
2378-1A	0	12451	13485	16007	12525	16017	12528	3300	15525	10675	14167	13015	12777	1744	12958	12407	1711	1744	12957
2381-2C		0	8242	16009	11996	16019	11999	12454	15492	11615	14119	12382	11669	12068	12046	12001	12081	12070	12637
2501-2D			0	16334	13240	16342	13237	13879	16044	13059	14668	13548	13097	13307	12349	13143	13318	13305	13890
2508-1C				0	16192	56	16197	14323	17156	17061	15334	16014	15736	15878	15949	16146	15898	15880	15775
3126-2A					0	16200	9	12547	15725	12407	14314	12900	12238	12146	12873	673	12157	12148	12812
3128-1B						0	16205	14337	17168	17067	15348	16024	15750	15888	15957	16154	15908	15890	15779
3137-2D							0	12550	15724	12410	14317	12901	12243	12149	12876	676	12160	12149	12815
3140-2B								0	15861	11209	14188	13049	12729	1660	13159	12394	1615	1660	12827
814-1A									0	11653	16426	15842	15349	15676	15726	15663	15683	15676	15441
820-1A										0	15573	14552	14403	10816	14021	12036	10831	10820	14623
822-2A											0	14257	14028	14003	14282	14228	14018	14005	14238
824-1A												0	12605	12602	12757	12761	12617	12602	12792
825-1C													0	12328	12481	12179	12345	12330	12451
827-2B														0	12636	11992	77	10	12521
828-1B															0	12813	12647	12636	6972
832-1A																0	12005	11992	12765
835-1B																	0	77	12532
837-2A																		0	12521
841-1C																			0
